# Median Facial Cleft in Amniotic Band Syndrome

**DOI:** 10.4103/0974-9233.80713

**Published:** 2011

**Authors:** Debabrata Das, Gobinda Das, Sibnath Gayen, Arpita Konar

**Affiliations:** Department of Ophthalmology, R.G. Kar Medical College, Kolkata 700 004, India; 1 Department of Pediatrics, R.G. Kar Medical College, Kolkata 700 004, India

**Keywords:** Amniotic Band, Bifid Nose, Median Facial Cleft, Syndactyly

## Abstract

Amniotic band syndrome manifests at birth with a variety of malformations ranging from constriction ring to defects incompatible to life, in various parts of the body. Although some theories have been proposed for the development of this syndrome, the exact cause remains unknown. The median facial cleft is an extremely rare manifestation of amniotic band syndrome with a relative paucity of reports available in the literature. Here, we report one such case.

## INTRODUCTION

Amniotic band syndrome (ABS) is a spectrum of asymmetrical congenital malformations due to ring-like constrictive bands in the limbs, head, face, or occasionally the trunk.^1^ The incidence of this syndrome ranges from 1:1200 to 1:15,000 live births.^2^ Nearly all cases are sporadic, and cases of familial transmission are rare.^3^ The exact etiology of the syndrome remains unknown. In 1930, Streeter proposed the “intrinsic theory” of germ defects in the embryonic disc as well as the amniotic cavity. However, the widely accepted “extrinsic theory” proposed by Torpin and Faulkner (1965) hypothesizes separation of amnion from the choroin during early pregnancy which produces free-floating tissue bands.^1^ These amniotic bands either wrap around parts of the embryo in a band-like manner or are swallowed by the fetus. The bands restrict growth or cause structural defects in the fetus.^4^ These bands are also the most frequent cause of facial clefts by creating pressure necrosis or interruption of normal fusion in the facial processes.^5^ We report a case of ABS with median facial cleft.

## CASE REPORT

**Figure 1:**
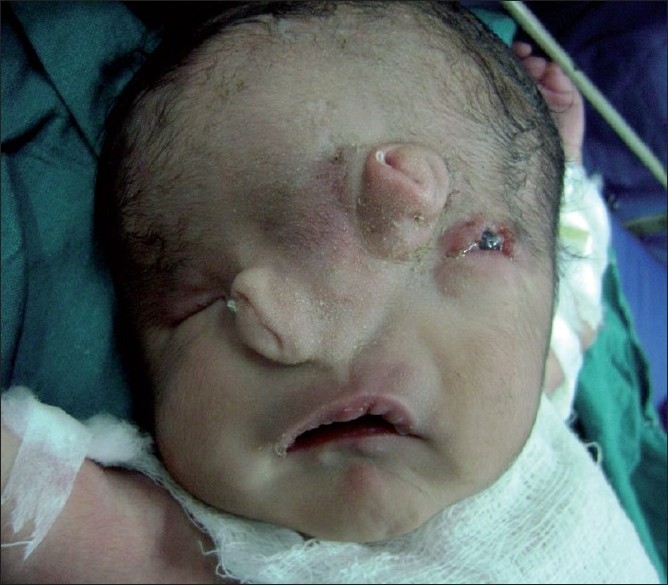
Clinical photograph of the face showing midline facial cleft with hypertelorism and bifid nose

**Figure 2:**
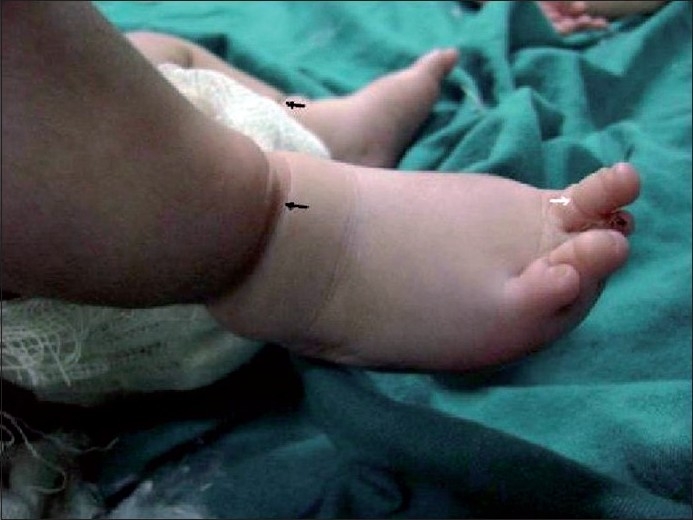
Clinical photograph of both legs with amniotic band (black arrows) and right second toe showing constricting bands (white arrows)

A 1-day-old male neonate presented with respiratory distress and bifid nose. The baby was the first born to a 24-year-old mother and was delivered full term through normal vaginal birth. His birth weight was 3.0 kg. Maternal obstetric history was not suggestive of any antenatal insult to the fetus. Family history revealed no consanguineous marriage or history of congenital malformation. On gross inspection of the face, there was a bifid nose with severely stenotic nostrils, cleft palate, hypertelorism, a microphthalmic right eye, and coloboma in the left upper lid [Figure 1]. Probing of the nostrils revealed a small cul de sac. The baby had constrictive circumferential grooves in both lower limbs and right second toe [Figure 2]. The right foot had amputated great toe. The left hand showed syndactyly of the fourth and fifth fingers. No other abnormalities were found on routine systemic clinical examination. Initial stabilization was performed via orogastric aspiration, and maintenance of airway and oxygenation. We planned a multidisciplinary surgical approach for reconstruction of the face; however, the parents left the hospital with the baby on a risk bond.

## DISCUSSION

Most of the cases of ABS are sporadic with no recurrence in siblings or children of the affected adults.^1^ Though exact etiopathogenesis of band formation is still unknown maternal trauma, oophorectomy during pregnancy, intrauterine contraceptive device, amniocentesis, and familial connective tissue disorders are some of the suggested risk factors for the development of ABS.^1^ These bands produce varying degrees of polymorphic clinical findings ranging from superficial circumferential grooves in the skin to amputation of part or whole of a limb. The morphogenesis of facial clefting in ABS is not well understood and is a topic of much debate. The multiple, polymorphic, and asymmetrical facial malformations are may be due to swallowing of amniotic bands by the fetus.^6^ Johnston^7^ and Wetson,^8^ however, suggested failure of migration or degeneration of neural crest cells are responsible for cleft formation.

Tessier in 1976 had simplified cranio-facial clefts classification from 0 to 14, beginning at 0 in the midline of the lower face and progressing clockwise around the right orbit ending at 14 in the midline of upper face that has been universally accepted.^9^ Losee *et al*. in 2004 simplified and classified congenital nasal abnormalities into only four types.^10^ Median facial cleft syndrome is included in Type III and results due to failure of fusion of the medial nasal processes. Median facial cleft corresponds with Tessier cleft No. 0 or 14 and Tessier cleft No. 9, 10, 11 are associated with upper lid coloboma. The upper lid coloboma may be the result of amniotic band traction on the lids. The malformations of ABS are summarized in Table 1.^2^,^5^ ABS should be included in the differential diagnosis of all complex asymmetrical malformations of extremities, face and body wall, e.g., amniotic folds, short umbilical cord syndrome, and extra-amniotic pregnancy.

**Table 1:** Anatomical and clinical malformations associated with amniotic band syndrome

Extremities–soft tissue constriction rings - Shortening of limb - Amputation of digits and toes - Syndactyly - Hypoplasia of digits - Foot deformities-club foot - Pseudoarthrosis - Peripheral nerve palsy Craniofacial–asymmetric face defects - Cleft lip and palate - Orbital defects (anophthalmos, microphthalmos, enophthalmos) - Corneal abnormalities, lid colobomas, defect of nose - Central nervous system–anencephaly, encephalocoele, meningocoele Abdomen–organ exstrophy, gastroschisis, omphalocele, imperforate anus Chest wall–heart exstrophy

In our case, the baby had Tessier cleft No. 0 and 10 with constricting bands in both lower limbs and the right second toe with autoamputation of the great toe and syndactyly of the left hand.

As neonates are nasal breathers, simultaneous sucking and breathing lead to respiratory distress. Temporary measures such as oral airway and orogastric feeding are successful for neonates. Canalization of the nasal passage and tracheostomy are other options on the basis of the severity of neonatal respiratory distress. Surgical reconstruction is very demanding and requires a multidisciplinary approach.
